# Strategies to accelerate cervical cancer elimination in Greece: a modeling study

**DOI:** 10.3389/fonc.2025.1480942

**Published:** 2025-05-27

**Authors:** Cody Palmer, Anastasios Skroumpelos, Ugne Sabale, Ilias Gountas, Georgios Trimis, Antonis Karokis, Theodoros Agorastos

**Affiliations:** ^1^ Health Economic and Decision Sciences, Merck & Co., Inc., Rahway, NJ, United States; ^2^ External Affairs, MSD, Athens, Greece; ^3^ Value & Implementation Outcomes Research, MSD, Vilnius, Lithuania; ^4^ Medical Affairs, MSD, Athens, Greece; ^5^ Department of Obstetrics and Gynecology, Aristotle University of Thessaloniki, Thessaloniki, Greece

**Keywords:** cervical cancer, human papillomavirus vaccine, HPV, HPV DNA, screening, nonavalent, 9-valent, Greece

## Abstract

**Introduction:**

Most cervical cancer cases are caused by human papillomavirus (HPV), a vaccine-preventable infection. According to the World Health Organization (WHO), both high HPV vaccination coverage and cervical cancer screening rates will accelerate the elimination of cervical cancer, a threshold defined as <4 age-standardized cases per 100,000 women.

**Methods:**

A dynamic transmission model was used to study the effect of increased HPV vaccination coverage and cervical cancer screening rates in Greece on cervical cancer incidence over a 100-year time horizon. Greek-specific or proxy data were used for both model inputs and calibration prior to the evaluation of eight different vaccination and screening scenarios. The estimated time to cervical cancer elimination and eradication in Greece was reported as the year each scenario reached <4 cases per 100,000 and <1 case per 100,000, respectively.

**Results:**

Greece reached the WHO cervical cancer elimination threshold by 2074 with a 50% HPV vaccination coverage and 50% Pap test screening rate. When HPV DNA-based methods replaced Pap tests at the same rate and HPV vaccination coverage levels, the WHO threshold was reached by 2061. Other scenarios modeled future changes in HPV DNA-based screening rates with either 50% or 90% vaccination coverage. The 75% HPV DNA-based screening with 90% vaccination coverage scenario reached the WHO threshold by 2047 and the eradication threshold before the end of the century (2096).

**Conclusion:**

If public health interventions are implemented to accelerate HPV vaccination coverage and HPV DNA-based screening adherence within the next five years, Greece can reach the WHO’s cervical cancer elimination threshold by 2047 and eradicate cervical cancer before the end of the century.

## 1 Introduction

Human papillomavirus (HPV) is a sexually transmitted virus that infects an estimated 8 out of 10 individuals during their lifetime (1). Persistent HPV infection may result in genital warts or a variety of anogenital and oropharyngeal cancers. There are 13.3 age-standardized cases of cervical cancer per 100,000 women worldwide, virtually all caused by HPV (2, 3). In 2020, it was estimated that over 600,000 women are diagnosed and over 340,000 women die annually due to HPV-related cervical cancer (4). To prevent HPV-related cancers, the World Health Organization (WHO) recommends that children receive an HPV vaccine prior to sexual debut between the ages of 9–14 (5).

In 2020, the WHO developed a strategy to accelerate the elimination of cervical cancer as a public health problem, particularly focusing on low- and middle-income countries (2). This strategy outlines specific goals for all countries to meet by 2030, including vaccinating 90% of girls by 15 years of age, screening 70% of women at least twice with a high-performance test by the ages 35 and 45, and providing access to treatment for at least 90% of women with precancerous lesions and 90% of women with invasive cancer (2, 5). If vaccination, screening, and treatment targets are met worldwide by 2030, it is estimated that the age-standardized incidence of cervical cancer will be on the path to fall below 4 cases per 100,000, the WHO’s threshold for cervical cancer elimination, within the next century. This reduction in cervical cancer incidence will prevent 60 million cases of cervical cancer and 45 million deaths worldwide by 2120 (2). The European Commission released “Europe’s Beating Cancer Plan” in 2022, renewing a commitment to cancer prevention, treatment, and care for individuals in the European Union (EU) (6). In June 2024, the European Council recommended that member states enhance country-specific efforts to increase HPV vaccination and cervical cancer screening rates. The European Council’s recommendation was for member states to reach a 90% vaccination rate of girls and to significantly increase vaccination coverage of boys by 2030, aligning with the WHO’s global initiative (2, 7).

The 9-valent HPV vaccine (Gardasil 9, Merck & Co., Inc. Rahway, NJ, USA) available in Greece, provides protection against seven high-risk HPV genotypes (16, 18, 31, 33, 45, 52, and 58) responsible for 90% of cervical cancers, as well as two low-risk HPV genotypes (6 and 11) that cause 85% of genital warts (3, 8). HPV infection may cause cervical intraepithelial neoplasia (CIN) that if left untreated can progress to invasive cervical cancer (3). For over 50 years, cervical cancer screening has used cytology-based methods, Pap tests, to detect abnormal cells in the cervix. These tests unfortunately have low sensitivity and poor reproducibility; however, the development of HPV DNA-based screening has increased the sensitivity of cervical cancer screening (9–11). Additionally, DNA-based methods enable women to be screened less frequently, every five years instead of every three years for Pap tests (9, 11). Several studies have modeled the impact of enhanced HPV vaccination rates and cervical cancer screening methods on reducing the incidence of cervical cancer to levels below the WHO’s cervical cancer elimination threshold (12–15). According to these studies, the elimination of cervical cancer could occur as early as 2028 for Australia, 2035 for Norway, and 2028 for the United States (12–14).

In Greece, the age-standardized incidence of cervical cancer is close to 8 cases per 100,000 women, comparable to the average age-standardized rate of other high-income countries worldwide (4, 16–18). The HPV vaccine has been approved by the Greek Republic Ministry of Health since 2008, and the estimated vaccination coverage rates for females 11–14 and 11–18 years of age are 43.8% and 55.4%, respectively, using 2019–2021 vaccine prescription data from the Greek healthcare national database, HDIKA (19, 20). In 2023, the national childhood vaccination program was updated to include a two-dose series of the 9-valent HPV vaccine for children aged 9–14 and a three-dose series for children aged 15–18 without copayment (8). In addition to low estimates of HPV vaccination coverage, the percentage of women receiving regular cervical cancer screening in Greece is unclear. A survey-based study from 2014 indicated that approximately 30.3% of Greek women consistently adhered to the recommendation of an annual Pap test, while another modeling study indicated a 39% cervical screening coverage rate among Greek women (15).

The limited data available indicate a need to increase both HPV vaccination rates and HPV DNA-based screening measures in Greece, both of which have been shown to be cost-effective preventive public health measures (11, 21–24). In July 2022, the Greek Parliament initiated a pilot cervical cancer screening program (Pap tests every three years for women 21–29 years of age and HPV DNA-based methods every five years for women 30–65 years of age), which will be funded through the Greek Recovery and Resilience Fund until 2025 (25). This program aspires to become the foundation for a future national cervical cancer screening program in Greece (25).

The Greek government has announced its commitment to preventive public health measures to lower the incidence of cervical cancer in Greece (25). The objective of this study was to use an established dynamic transmission model to assess the timeframe for Greece to reach cervical cancer elimination (<4 cases per 100,000) and eradication (<1 case per 100,000) with different HPV vaccination and screening scenarios. The findings of this study may help establish a timeline for the elimination of cervical cancer in Greece, thereby informing pertinent public health policy objectives aimed at eliminating and eradicating the disease.

## 2 Methods

### 2.1 Model overview

**Figure 1:**
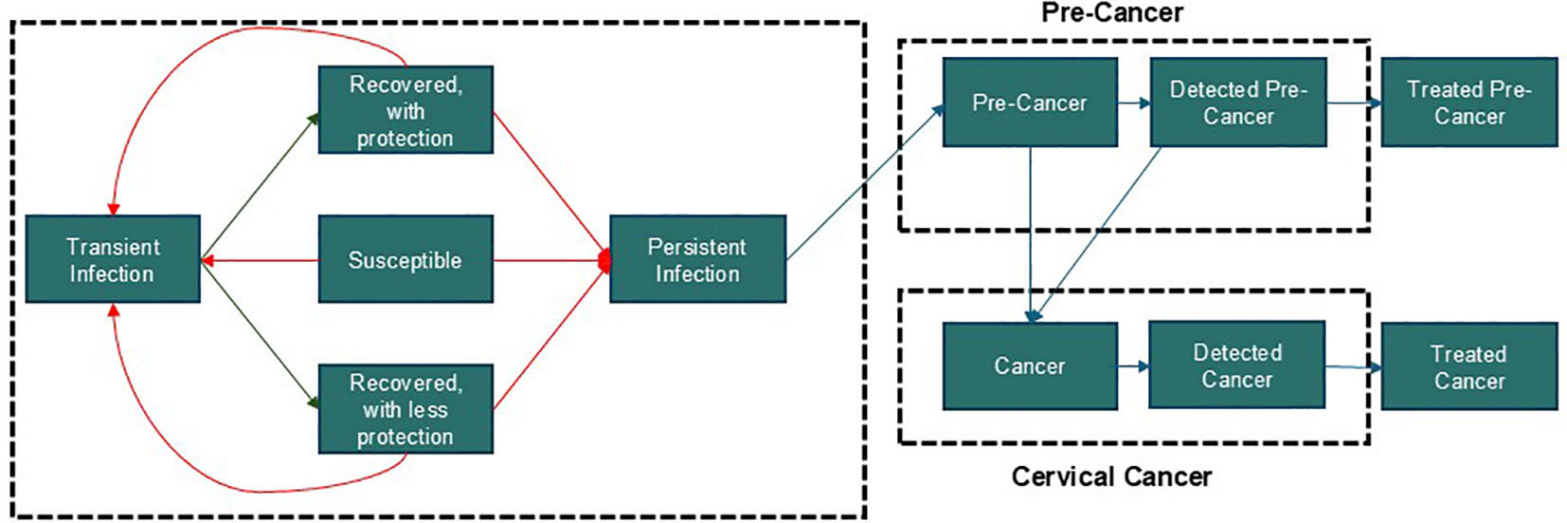
Study model ^A^. Dynamic model of HPV transmission adapted for Greece (3, 26–29). The model is structured by age, sex, sexual activity, and incorporates both direct and indirect herd immunity effects of vaccination. These features are fundamental to HPV transmission dynamics and intervention strategies, making them essential components of our model. This particular structure was chosen to effectively capture these key facets while maintaining analytical clarity. While more computationally sophisticated models, such as agent-based models, could be employed, their added value for this specific analysis would be marginal. Our model strikes an optimal balance between capturing critical epidemiological details and ensuring computational efficiency.

A dynamic transmission model was adapted to determine the effect of increased 9-valent HPV vaccination coverage for girls 10–14 years of age and cervical cancer screening on the age-standardized incidence of cervical cancer over a horizon of 100 years (2024–2124) in Greece. It was a continuous, population-based, compartmental, deterministic model that has been described in previous analyses (**Figure 1**) (3, 26–29). The model was structured by age, sex, and sexual activity, and incorporated both direct and indirect herd immunity effects of vaccination (**Supplementary Figure 1**). Greek-specific data or proxy data was collected from January 1, 2011 to December 31, 2011 for model calibration.

### 2.2 Model inputs, parameters, and calibration

#### 2.2.1 Demographics

The model projected Greek demographics using age group and all-cause mortality data derived from the United Nations’ World Population Prospects (**Supplementary Table 1**; 30) with the method described by Hethcote et al. (31). The proportion of males and females in each age group were graphed comparing both the model and actual Greek population data for 2011 (**Supplementary Figure 2**) (30).

#### 2.2.2 Sexual behavior

Sexual behavior data for the UK used in a recent adaptation of this model was used as a proxy due to the absence of sufficient sexual behavior data for Greece in the literature (3). Since a proxy was used, a Greek-specific correction factor for the number of partners was incorporated during the calibration of the model. The factor did not depend on age or sex and was computed through calibration where it, along with other parameter values, were varied to find a best fit to Greek-specific data.

#### 2.2.3 Cervical cancer screening, treatment for precancer and cervical disease

In general, treatment for HPV-related precancers depends upon early detection by cervical cancer screening. A 2011 survey of women in Greece found that 12.4% of women never had a Pap test, 44.8% had regular Pap tests, and 30.3% had annual Pap tests for five consecutive years (32). With this in mind, the population was divided into two groups: women who were regularly screened at a rate of once per year, and women who were screened irregularly, never screened, or screened less than once per year. The age-specific screening rates for the latter group were calculated through calibration methods as in recent models (**Supplementary Table 2**) (3, 26, 29).

The performance of screening tests to detect cervical cancer was assumed to be equivalent to previous sensitivity and specificity data for Pap and colposcopy tests (3). In scenarios involving DNA screening, the sensitivity of the test was assumed to be 95% for HPV-infected women based on values reported from previous studies and test manufacturers, aligning with the performance of these tests on CIN levels, including CIN2 and CIN3 (CIN2+) (13). After an abnormal screening test, it was assumed that 90% of women would seek a follow-up test and treatment based on the goals of the 2020 WHO Global Elimination Strategy for cervical cancer (2). Additionally, the model assumed that 50% of women were treated after a CIN1 diagnosis and 100% were treated after a CIN2+ CIN3 diagnosis (26). The rate of invasive cervical cancer symptom recognition by women was also included from a previously validated model adaptation (3). Lastly, it was assumed that women who received a hysterectomy would not have any level of cervical disease, and any cervical disease would be cleared at the time of the hysterectomy. Hysterectomy rates from the UK were used as a proxy due to the absence of data for Greek women (33).

#### 2.2.4 Historical HPV vaccination in Greece

Greece added HPV vaccination for girls in 2008; however, since the program is not school-based, coverage has remained low (34). The estimated vaccination coverage rates for females 11–14 and 11–18 years of age were 43.8% and 55.4%, respectively, using 2019–2021 data on vaccine prescriptions from the Greek healthcare database HDIKA (19, 20). Based on this data, a vaccination coverage rate of 50% was chosen as the input value of baseline HPV vaccination coverage in Greece. This model used an age range of 10–14 years of age for HPV vaccination, which is still considered an age range prior to sexual debut.

#### 2.2.5 HPV prevalence in Greece

Baseline HPV genotype (16, 18, 31, 33, 45, 53, and 58) prevalence data for each age group (14–25, 26–46, and ≥47) were computed by multiplying the prevalence of each HPV genotype in 2011 by the total HPV prevalence in 2011 for each age group (35). For the model, it was assumed that these 2011 HPV prevalence values estimated the pre-vaccination HPV infection burden in Greece (**Supplementary Table 3**, **Supplementary Figure 3**).

#### 2.2.6 Cervical cancer incidence in Greece

Cervical cancer incidence data for different age groups in Greece was obtained from the Catalan Institute of Oncology (ICO) and the International Agency for Research on Cancer (IARC) HPV Information Center and the target age-standardized cervical cancer incidence used was 8.05 cases per 100,000 women (**Supplementary Table 4**) (16).

#### 2.2.7 HPV genotype attributable fraction in Greece

The fraction of cervical cancers attributed to each HPV genotype was based on a meta-analysis of invasive cervical cancer cases (**Supplementary Table 5**) (16). It was assumed that all cervical cancers were caused by high-risk HPV, thus the fraction attributable to other HPV types includes all genotypes outside of the seven high-risk types covered by the vaccine. The combined HPV genotypes 45, 52, and 58 attributable fraction of 6% was selected using data on the prevalence of these HPV genotypes in Europe from 2010 as well as the confidence interval from the ICO/IARC HPV Information Center report (16, 36). Since vaccine efficacy against each HPV type is assumed to be the same, the distribution of the 6% among the three genotypes was inconsequential (**Supplementary Table 5**). Additional HPV-type-specific parameters included in the model were: transmission probability and contact correction (males and females), clearance of transient infections (males and females), degree of immunity to subsequent infection after seroconversion (males and females), proportion of female infections that progressed to invasive cervical disease, rate at which HPV infections in women progressed to CIN1, 2, or 3, and the proportion of individuals who seroconverted after clearing a transient infection (males and females; **Supplementary Table 6**).

### 2.3 Cervical cancer screening and vaccination coverage rate scenarios

Outputs from the model included both the age-standardized incidence and elimination year of cervical cancer if eight different vaccination and screening scenarios were implemented in Greece within five years (**Table 1**). The elimination year was defined as the year when cervical cancer incidence dropped to <4 cases per 100,000 women (WHO’s elimination threshold) and an aspirational eradication threshold of <1 case per 100,000 women (2, 12). Scenarios 1 and 2 were representative of the previous status quo in Greece, Pap screening (30.3% or 50%, respectively) and 50% HPV vaccination coverage (25). The pre-2022 regular cervical screening status quo of 30.3% was selected from a 2014 survey-based study of women in Greece (32). The 50% screening level was selected for scenario 2 after expert opinion since the 30.3% screening adherence was reported during a time of economic crisis in Greece, and there have not been additional studies looking at the impact of an improved economic environment on screening adherence levels (37). Scenarios 3 and 4 included 50% HPV vaccination coverage and Pap screening was replaced with HPV DNA-based screening (30.3% or 50%, respectively), representing the current status quo since the introduction of the 2022 pilot cervical screening program in Greece (25). Scenarios 5–8 represented future policy options that focus on increased vaccination rates up to 90% and increased cervical screening adherence up to 75%. The 75% screening adherence target was selected since it is in the middle of the upper tier of cervical cancer screening programs in Europe from 2021 (70%–80%) (38). For scenarios 3–8, regular screening adherence is defined as a HPV DNA-based test once every five years (25). Additionally, it was assumed that there was a ten-year transition from Pap tests to HPV DNA-based tests with the proportion of Pap tests decreasing linearly over time.

**Table 1:** Estimated elimination year of cervical cancer in Greece under different screening and HPV vaccination scenarios ^A,B^.

Scenario	Type of screening	Screening rate	Vaccination coverage rate	Cervical cancer incidence ^C^		
				<4 cases/100,000^D^	<2 cases/100,000	<1 case/100,000
1^E^	Pap	30.30%	50%	>2125	>2125	>2125
2^E^	Pap	50%	50%	2074	>2125	>2125
3^F^	HPV DNA	30.30%	50%	2079	>2125	>2125
4^F^	HPV DNA	50%	50%	2061	>2125	>2125
5^G^	HPV DNA	30.30%	90%	2068	2090	2117
6^G^	HPV DNA	50%	90%	2057	2083	2104
7^G^	HPV DNA	75%	50%	2047	2092	>2125
8^G^	HPV DNA	75%	90%	2047	2076	2096

^A^30.3% is the estimated baseline cervical cancer screening rate derived from a 2011 survey of Greek woman (32). The additional cervical screening rates of 50% and 75% were from derived from expert opinion and aspirational rates described in the methods.

^B^The 50% vaccination coverage rate was based on the estimated baseline vaccination coverage for Greek females 11–14 years of age (43.8%) and females 11–18 years of age (55.4%) from 2019–2021 using data on vaccine prescriptions from the Greek healthcare database, HDIKA (19, 20). The 90% vaccination rate was derived from WHO recommended guidelines from 2020 (2).

^C^Reported as age-standardized cervical cancer incidence.

^D^World Health Organizations’ threshold for the elimination of cervical cancer as a public health problem (2).

^E^Scenarios 1 and 2 model traditional Pap screening and vaccination coverage rates of girls on cervical cancer incidence in Greece (32).

^F^Scenarios 3 and 4 model the effect of the change in cervical cancer screening program 2022 to HPV DNA-based screening methods on cervical cancer incidence in Greece (25).

^G^Scenarios 5–8 model the effect of higher HPV DNA-based screening rates and higher vaccination coverage rates of girls on cervical cancer incidence in Greece, if policies in Greece are changed within 5 years.

## 3 Results

### 3.1 Model fit

Overall, the model achieved a good fit with actual Greek population data (**Supplementary Figures 2–4**). The model’s estimated age-standardized incidence of cervical cancer was 8.45 per 100,000 women, compared with the actual age-standardized incidence of 8.05 per 100,000 women in Greece (16).

### 3.2 Scenario analyses

**Figure 2:**
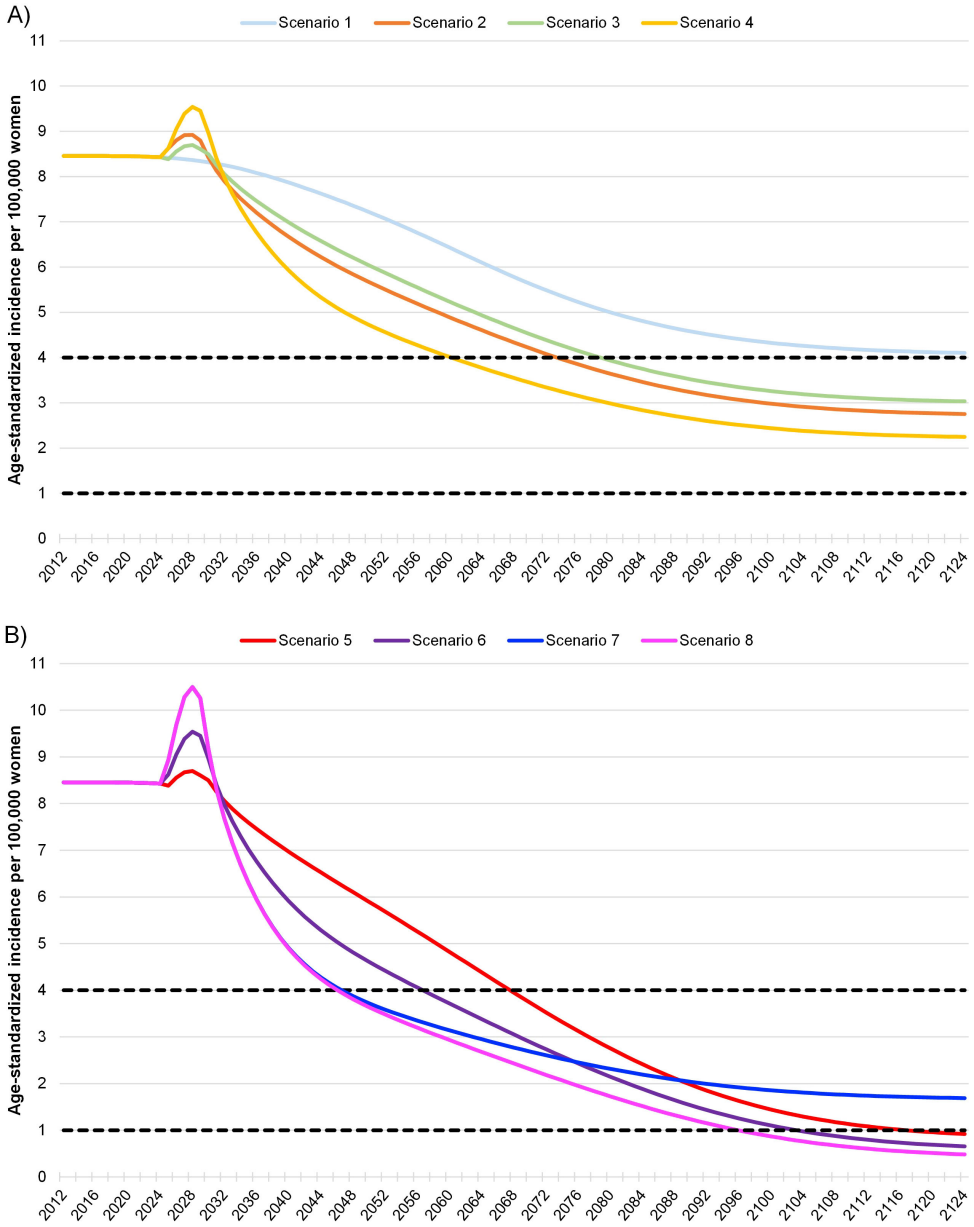
Model of cervical cancer incidence in Greece under different screening and vaccination coverage scenarios ^A^. The dashed lines indicate both the World Health Organizations’ threshold of 4 cases per 100,000 for the elimination of cervical cancer as a public health problem and an aspirational threshold of 1 case per 100,000 (2). For HPV DNA-based testing, it was assumed that there was a ten-year transition from Pap tests to HPV DNA-based methods with the proportion of Pap tests decreasing linearly over time. The start of the 100-year horizon is in 2024. **(A)** Scenarios 1–2 represent the previous status quo of Pap screening prior to 2022 cervical cancer screening guideline changes in Greece. Scenarios 3–4 represent the current status quo of HPV DNA-based screening after the cervical cancer screening pilot program was introduced by the Greek Parliament in 2022 (25). Scenarios 1–4 all assume a 50% vaccination rate for the Greek population. **(B)** Scenarios 5–8 represent aspirational future increases to both cervical cancer screening and vaccination guidelines in Greece.

Eight different scenarios were examined in the dynamic transmission model, as shown in **Table 1**. Analysis of the scenarios representing the previous status quo (scenarios 1 and 2) revealed that achieving the elimination of cervical cancer or an incidence of <4 cases per 100,000 would be feasible by 2074 at the earliest (scenario 2; **Table 1**). Subsequently, the incidence would decline to around 3 cases per 100,000 and remain relatively stable thereafter (**Figure 2A**). A transition to HPV DNA-based screening with similar levels of screening adherence and vaccination coverage resulted in elimination occurring earlier by 2061 for scenario 4 (**Table 1**). Furthermore, cervical cancer incidence would continue to decline until reaching a level between 3 and 2 cases per 100,000 for the remaining years of the model’s time horizon (**Figure 2A**).

Increasing vaccination coverage to the WHO recommended level of 90% led to cervical cancer elimination by 2068 or 2057, depending on the HPV DNA-based screening rate (scenarios 5 and 6, **Table 1**). Additionally, the decline in cervical cancer incidence for scenarios 5 and 6 was steeper compared to that of scenarios 1 through 4, resulting in <1 case per 100,000 by 2117 or 2104, and continuing to decrease until 2125 (**Figures 2A, B**). Alternatively, maintaining the current vaccination coverage of 50% and increasing HPV DNA-based screening adherence to 75% (scenario 7) moved the elimination date up to 2047. However, the aspiration of <1 case per 100,000 was not achieved within the model’s time horizon (**Table 1**, **Figure 2B**).

A dual-focus strategy with 90% vaccination coverage and 75% adherence to HPV DNA-based screening for scenario 8 was estimated to have the same elimination year of 2047 as scenario 7 (**Table 1**). However, cervical cancer incidence continued to decrease for scenario 8, reaching <2 and <1 case per 100,000 by 2076 and 2096, respectively (**Table 1**). This decline continued to 0.48 cases per 100,000 within the model’s time horizon (**Figure 2B**). Notably, scenario 8 with 75% HPV DNA-based screening and 90% vaccination coverage, would reach half the WHO target incidence (<2 cases per 100,000 around 2076, close to the year scenario 2 (previous status quo scenario) and would approach the <4 cases per 100,000 threshold (2074, **Table 1**).

## 4 Discussion

Both preventive measures, HPV DNA-based tests and HPV vaccinations, plan crucial yet distinct roles for the elimination of cervical cancer. Cervical cancer screening immediately detects precancerous lesions or HPV infection, lowering the incidence of cervical cancer after preventive treatment, while HPV vaccination prevents HPV infection that may lead to cervical cancer 15–20 years later (39). This study’s model indicates that increasing cervical cancer screening adherence has a direct and immediate impact on the time to cervical cancer elimination in Greece. If quinquennial HPV DNA-based screening adherence levels increase to 75% within the next five years, Greece will be able to eliminate cervical cancer as a public health problem (<4 cases per 100,000 women) within 23 years (by 2047). Additionally, this study validates a switch from Pap test screening to HPV DNA-based screening as an effective and superior public health strategy for Greece (11). Scenarios 3 and 4 with HPV DNA-based testing reached the WHO elimination threshold by 2079 and 2061, respectively more than 45 or 13 years earlier than matching scenarios with Pap test screening (scenarios 1 and 2).

The importance of HPV DNA-based cervical screening and HPV vaccination as dual preventive strategies is illustrated in scenarios 7 and 8, which have the same cervical cancer elimination year of 2047 despite differences in vaccination coverage. Scenarios 7 and 8 have the highest HPV DNA-based screening rate of 75%, allowing both scenarios to reach <4 cases per 100,000 by 2047; however, the delayed impact from 90% HPV vaccination coverage for scenario 8 causes an additional steep decline in cervical cancer incidence to <2 cases per 100,000 by 2076 and <1 case per 100,000 by 2096. This finding aligns with results from a US cervical cancer modeling study which showed the importance of increasing both screening and vaccination coverage to expedite the elimination of cervical cancer (12).

This study is the first to analyze the impact of a variety of increased HPV vaccination and cervical cancer screening scenarios on cervical cancer incidence in Greece. A study modeling the WHO guidelines for the elimination of cervical cancer for multiple countries worldwide predicted Greece would reach <4 cases per 100,000 women between 2045 and 2050 (15). This current study identified a similar timeframe for cervical cancer elimination with 75% screening adherence and 50% HPV vaccination coverage. It is also important to note that all scenarios with a 90% HPV vaccination rate, in alignment with the WHO guidelines, were the only ones able to reduce cervical cancer incidence to the aspirational goal of <1 case per 100,000 within the model’s time horizon, regardless of cervical cancer screening adherence rate (2). Hence, cervical cancer prevention policies in Greece should focus on increasing both vaccination and screening adherence since each measure is equally important and non-interchangeable in the pursuit of cervical cancer elimination in a timely manner. Additionally, Greece is considered to be a highly developed country, so it is expected that screening adherence should be greater than only two cervical cancer screening tests within a lifetime as recommended by the WHO (18). However, challenges for highly developed countries include failure from non-participation, underscreening, and lack of follow-up after abnormal results (9). Therefore, it is necessary to facilitate increased adherence to a new national HPV vaccination and screening program in Greece with targeted public health measures.

Other countries such as the US, Norway, Sweden, and Australia have examined the impact of screening adherence levels with their national cervical cancer screening programs (12, 13, 28, 40). It was estimated using a dynamic transmission model that Australia could eliminate cervical cancer by 2035 with increased vaccination and screening measures (13). In Australia, monitoring by the National Health and Medical Research Council (NHMRC) Center of Research Excellence in Cervical Cancer Control database indicates that the cervical cancer screening rate is the lowest for rural populations (41). Self-sampling, where women collect a sample for a HPV DNA-based test themselves, has been proposed as a strategy to reach populations living in remote areas (13). A 2019 meta-analysis of studies comparing self-sampling with traditional cervical screening methods worldwide showed that women were twice as likely to participate in screening programs using this method (42). Self-sampling was also implemented in Sweden during the COVID-19 pandemic, and the increase in screening rates to 85% resulted in a long-term change to Sweden’s screening program (40). A recent study in Greece looked at self-sampling in rural areas using a well-established midwifery network to provide screening kits and a targeted community information campaign by local physicians. The results showed it was feasible to achieve higher screening rates using this method when other measures to reach eligible patient populations are not easy to implement (43, 44). Additionally, women who were HPV positive after self-sampling had a high compliance to colposcopy referral rate ranging from 68.6% (for women 25–29) to 76.3% (for women 40–49), indicating this screening method may aid the early detection and prevention of cervical cancer for these populations (44).

Besides innovative self-sampling screening measures, an increase in public health outreach, a national data system to record patient information, and an adequate and sustainable funding source are necessary to increase both cervical cancer screening and HPV vaccination rates in Greece (6, 41, 45). In Sweden, instead of a national public information campaign, targeted communication occurs to high-risk patients identified with an electronic health records database (40). This cost-effective strategy has allowed Sweden to catch up and surpass other country’s HPV vaccination and cervical cancer screening rates, and it may be the first country to eliminate cervical cancer by 2030 (40). Sweden’s cervical cancer screening levels in 2021 reached 85% and HPV vaccination coverage for girls and boys was 90% and 85%, respectively (40). Greece should look toward Sweden’s accomplishment when deciding how to enhance its own public health communication efforts. Besides implementing targeted communication, it is also important to focus efforts on certain populations in Greece. For instance, healthcare professionals in Greece are an important source of information when parents decide to vaccinate their children against HPV. In fact, half of adults surveyed during a 2022 study in Greece said a physician’s recommendation led to their decision to have their child vaccinated with the HPV vaccine (46). Another study noted that Greek mothers who completed higher education were more likely to vaccinate their children with vaccines in the national vaccination program, including the HPV vaccine (47). These results point not only to the importance of physicians in communicating vaccination and screening information but also to the importance of targeted communication to mothers who have not completed higher education (47).

Overall, it is necessary for Greece to develop and maintain a comprehensive national healthcare database to monitor and report HPV incidence as well as vaccination and screening rates. This database will facilitate current and future public health measures in Greece. The establishment of an electronic health record database in all countries is one of the priority actions recommended by both the WHO and European Council to eliminate cervical cancer as a public health problem (2, 6, 7). The June 2024 European Council’s recommendations also asked member states, including Greece, to facilitate HPV vaccine uptake by administrating vaccines in both schools and pharmacies outside of physician offices, changing parental consent to an opt-out approach, and communicating evidence-based information about HPV vaccinations through a centralized source. Additionally, to enhance cervical cancer screening, the European Council recommends that Greece identifies a clear national screening target rate and that screening is easily available for all populations (7). The European Council’s recommendation indicates the availability of funding for member states to achieve these goals, including support for electronic database implementation and communication efforts (7). This support from the EU could complement Greece’s efforts toward the elimination of cervical cancer as a public health concern.

One limitation of this model is that in the absence of Greek-specific data, proxy data from various countries were used for certain model parameters. This is a common practice for model studies when country-specific data are unavailable (28). In this study, the proxy data decreased the quality of the fit of the model to the actual Greek HPV genotype prevalence data for females ≥47 years of age. The UK proxy data indicated that women ≥47 years of age were less sexually active than other age groups, which correlates with a lower HPV prevalence for that age group (3). Additionally, in general, older women have a lower incidence of HPV due to immunity from previous cleared infections (48). However, the actual Greek HPV incidence data for the ≥47 age group was higher than the model predictions (35). This difference did not impact the overall results since cervical cancer incidence was reported as an age-standardized value. In the present model, the 2011 HPV prevalence values were assumed to represent the pre-vaccination HPV infection burden in Greece. While the underlying HPV infection rate may change over time due to shifts in sexual behavior, modeling these future behavioral trends introduces substantial uncertainty. An additional limitation of this study is that it did not incorporate the impact of HPV vaccination of boys on cervical cancer. The purpose of this study however was to illustrate how public health measures aligned with the WHO recommendations (90% girls’ vaccination, 70% women screening, and 90% treatment) would impact the time to cervical cancer elimination in Greece. Future studies can determine what HPV vaccination rate of boys should be met in Greece to help decrease cervical cancer incidence even further. Finally, this study did not assess the cost-effectiveness of different screening and vaccination strategies. While these strategies have been found to be cost-effective in several other countries (11, 21–24), future studies on cost-effectiveness are needed in the Greek context.

## 5 Conclusion

In Greece, cervical cancer elimination, as defined by the WHO as <4 cases per 100,000 women, can be achieved by 2047 if high vaccination and cervical cancer screening rates are achieved within the next five years. The optimal target of 75% screening adherence and 90% HPV vaccination coverage are in line with the WHO recommendations for the elimination of cervical cancer within the next century (2). Considering the impact of these two primary interventions on cervical cancer prevention in Greece, the most effective means of ensuring optimal protection is to prioritize the vaccination of girls 9–18 years of age and to actively promote the screening of women 21–65 years of age. This comprehensive proactive approach will provide a robust public health strategy to safeguard families from experiencing cervical cancer now and in the future, securing intergenerational solidarity. The sooner policies are implemented in Greece, the greater number of cervical cancer cases and deaths will be averted.

## Data Availability

The original contributions presented in the study are included in the article/**Supplementary Material**. Further inquiries can be directed to the corresponding author.
