# Supplementary material for: Strategies to accelerate cervical cancer elimination in Greece: a modeling study
Source: Front Oncol. 2025 May 27;15:1480942. doi: 10.3389/fonc.2025.1480942 (PMC12148835; doi:10.3389/fonc.2025.1480942)
Supplement: Supplementary file 1 [file DataSheet1.docx]

## SUPPLEMENTARY MATERIALS

Supplemental Table 1. Annual all-cause mortality for the general population in Greece ^A^

| **Age group (years)** | **Annual all-cause mortality (per 100,000)** | |
| --- | --- | --- |
|  | **Males** | **Females** |
| 0-4 | 0.00358 | 0.00315 |
| 5-9 | 0.00053 | 0.00041 |
| 10-14 | 0.00043 | 0.00043 |
| 15-19 | 0.00133 | 0.00049 |
| 20-24 | 0.00264 | 0.00089 |
| 25-29 | 0.00340 | 0.00100 |
| 30-34 | 0.00456 | 0.00199 |
| 35-39 | 0.00500 | 0.00232 |
| 40-44 | 0.00755 | 0.00423 |
| 45-49 | 0.01494 | 0.00632 |
| 50-54 | 0.02201 | 0.01027 |
| 55-59 | 0.03922 | 0.01670 |
| 60-64 | 0.05270 | 0.02036 |
| 65-69 | 0.08170 | 0.03296 |
| 70-74 | 0.09876 | 0.04715 |
| 75-79 | 0.20123 | 0.12147 |
| 80-84 | 0.32693 | 0.25910 |
| ≥85 | 0.53496 | 0.48207 |

^A^ The all-cause mortality was calculated by dividing the number of deaths by the estimated mid-year population size for that age group in 2011 [[1](#_ENREF_1)].

Supplemental Table 2. Annual probability of irregular cervical cancer screening ^A^

| **Age group (years)** | **Annual probability of irregular screening** |
| --- | --- |
| 20-29 | 0.000 |
| 30-39 | 0.019 |
| 40-49 | 0.137 |
| 50-59 | 0.200 |

^A^ Data were calculated using methods from previous dynamic transmission models [[2-4](#_ENREF_2)].

Supplemental Table 3. HPV genotype prevalence in Greek females ^A^

| **HPV genotype** | **Age group (years)** | | |
| --- | --- | --- | --- |
|  | **14-25** | **26-46** | **≥47** |
| 16 | 5.6% | 5.0% | 4.1% |
| 18 | 1.2% | 1.1% | 0.9% |
| 31 | 2.6% | 2.3% | 1.9% |
| 33 | 0.6% | 0.6% | 0.5% |
| 45 | 0.6% | 0.6% | 0.5% |
| 52 | 1.6% | 1.4% | 1.2% |
| 58 | 2.7% | 2.4% | 2.0% |

^A^ Data were derived from Argyri et al [[5](#_ENREF_5)]. Baseline HPV genotype (16, 18, 31, 33, 45, 53 and 58) prevalence data for each age group (14-25, 26-46 and ≥47) were computed by multiplying the prevalence of each HPV genotypes in 2011 by the total HPV prevalence in 2011 for each age group. In order to account for the existing HPV infection rate prior to vaccination, we assumed the system had reached an epidemiological equilibrium—meaning that infection and disease levels were stable before vaccination was introduced. To simulate this, we ran the model with only screening in place (no vaccination) until outcomes stabilized. These steady-state values were then used as the base rates for HPV infection and cervical cancer prior to the start of vaccination.

Supplemental Table 4. Age-standardized cervical cancer incidence in Greece ^A^

| **Age group**  **(years)** | **Cervical cancer incidence**  **(per 100,000)** |
| --- | --- |
| 20-24 | 0.77 |
| 25-29 | 7.17 |
| 30-34 | 13.2 |
| 35-39 | 17.4 |
| 40-44 | 19.5 |
| 45-49 | 19.9 |
| 50-54 | 18.1 |
| 55-59 | 15.8 |
| 60-64 | 13.5 |
| 65-69 | 13.5 |
| 70-74 | 14.8 |
| 75-79 | 17.9 |
| 80-84 | 22.2 |
| ≥85 | 28.4 |

^A^ Table adapted from ICO/IARC Information Centre on HPV and Cancer report for Greece [[6](#_ENREF_6)].

Supplemental Table 5. Percentage of cervical cancers in Greece attributable to HPV genotypes ^A^

| **HPV genotype** | **Percentage of cervical cancers** |
| --- | --- |
| 16 | 45.1% |
| 18 | 33.0% |
| 31 | 3.8% |
| 33 | 4.4% |
| 45 | 2.0% |
| 52 | 2.0% |
| 58 | 2.0% |
| Other | 7.7% |

^A^ Table adapted from ICO/IARC Information Centre on HPV and Cancer report for Greece and Sanjose et al [[6](#_ENREF_6), [7](#_ENREF_7)].

Supplemental Table 6. Parameters of natural history of cervical disease ^A^

| **Parameter description** | **Genotype** | | | | | | | |
| --- | --- | --- | --- | --- | --- | --- | --- | --- |
|  | **16** | **18** | **31** | **33** | **45** | **52** | **58** | **Other** |
| **Clearance rate of transient infections** |  |  |  |  |  |  |  |  |
| Male | 1.2064 | 1.8495 | 1.1538 | 1.6164 | 1.2412 | 1.3588 | 1.1539 | 1.3595 |
| Female | 0.4946 | 0.7602 | 0.2745 | 0.4065 | 0.2747 | 0.2765 | 0.2745 | 0.6911 |
| **Transmission probability and contacts correction** |  |  |  |  |  |  |  |  |
| Male | 0.1344 | 0.3456 | 0.0571 | 0.1804 | 0.0206 | 0.0359 | 0.2640 | 0.1387 |
| Female | 1.1543 | 0.4461 | 0.8164 | 0.3267 | 1.0033 | 0.9521 | 0.2858 | 0.9470 |
| **Population clearing transient infections that seroconvert** |  |  |  |  |  |  |  |  |
| Male | 0.0012 | 0.0000 | 0.0000 | 0.0717 | 0.0011 | 0.0011 | 0.0210 | 0.0935 |
| Female | 0.4939 | 0.8118 | 0.9892 | 0.9269 | 0.7172 | 0.9370 | 0.9728 | 0.8151 |
| **Protection against subsequent infection after seroconversion** |  |  |  |  |  |  |  |  |
| Male | 0.0135 | 0.6075 | 0.8999 | 0.9099 | 0.2238 | 0.9498 | 0.0467 | 0.8037 |
| Female | 0.0002 | 0.4583 | 0.0000 | 0.9638 | 0.0494 | 0.0244 | 0.0285 | 0.5874 |
| **Infections that progress to cervical disease** | 0.2497 | 0.0834 | 0.0235 | 0.0145 | 0.0043 | 0.0031 | 0.0033 | 0.0082 |
| **Rate which persistent infections progress to CIN1** | 0.0024 | 0.0122 | 0.1077 | 0.2856 | 0.4303 | 0.1556 | 0.1155 | 0.1113 |
| **Rate which persistent infections progress to CIN2** | 0.0019 | 0.0122 | 0.0004 | 0.0809 | 0.3005 | 0.1140 | 0.0150 | 0.2213 |
| **Rate which persistent infections progress to CIN3** | 0.0018 | 0.0064 | 0.0000 | 0.0001 | 0.0990 | 0.0950 | 0.0000 | 0.0000 |

^A^ Parameters were determined by model calibration similar to previous studies [[2-4](#_ENREF_2)].

Supplemental Figure 1. Flow chart outlining the modeling process

Supplemental Figure 2. Proportion of the population in each age group ^A^

^A^ Baseline Greek population data were derived from the United Nations’ World Population Prospects. The model projected Greek demographics using age group and all-cause mortality data with the method described by Hethcote et al [[8](#_ENREF_8)].

Supplemental Figure 3. Model prediction versus known HPV genotype prevalence in Greek females ^A^

^A^ Baseline data values were calculated with HPV genotype-specific prevalence data from 2011 (see Supplemental Table 3) by the total HPV prevalence in 2011 for each age group [[5](#_ENREF_5)].

Supplemental Figure 4. Model prediction versus actual age-specific incidence of cervical cancer in Greece

References

1. United Nations: Department of Economic and Social Affairs. World Population Prospects 2022 2022. Available from: <https://population.un.org/wpp/Download/Standard/MostUsed/>.

2. Palmer C, Keisuke T, Machiko A, Elbasha E. Public health impact and cost effectiveness of routine and catch-up vaccination of girls and women with a nine-valent HPV vaccine in Japan: a model-based study. BMC Infectious Diseases. 2021;21:(1). 10.1186/s12879-020-05632-0.

3. Owusu-Edusei K, Palmer C, Ovcinnikova O, Favato G, Daniels V. Assessing the Health and Economic Outcomes of a 9-Valent HPV Vaccination Program in the United Kingdom. J Health Econ Outcomes Res. 2022;9:(1). 10.36469/001c.34721.

4. Daniels V, Prabhu VS, Palmer C, Samant S, Kothari S, Roberts C, et al. Public health impact and cost-effectiveness of catch-up 9-valent HPV vaccination of individuals through age 45 years in the United States. Human vaccines & immunotherapeutics. 2021;17:(7). 10.1080/21645515.2020.1852870.

5. Argyri E, Tsimplaki E, Papatheodorou D, Daskalopoulou D, Panotopoulou E. Recent Trends in HPV Infection and Type Distribution in Greece. Anticancer Res. 2018;38:(5). 10.21873/anticanres.12565.

6. Bruni L AG, Serrano B, Mena M, Collado JJ, Gómez D, Muñoz J, Bosch FX, de Sanjosé S. ICO/IARC Information Centre on HPV and Cancer (HPV Information Centre): Human Papillomavirus and Related Diseases in Greece 2023; cited 2023. Available from: <https://hpvcentre.net/datastatistics.php>.

7. de Sanjose S, Quint WG, Alemany L, Geraets DT, Klaustermeier JE, Lloveras B, et al. Human papillomavirus genotype attribution in invasive cervical cancer: a retrospective cross-sectional worldwide study. Lancet Oncol. 2010;11:(11). 10.1016/S1470-2045(10)70230-8.

8. Hethcote HW. An age-structured model for pertussis transmission. Math Biosci. 1997;145:(2). 10.1016/s0025-5564(97)00014-x.
